# Generation of Functional Beta-Like Cells from Human Exocrine Pancreas

**DOI:** 10.1371/journal.pone.0156204

**Published:** 2016-05-31

**Authors:** Maria J. Lima, Kenneth R. Muir, Hilary M. Docherty, Neil W. A. McGowan, Shareen Forbes, Yves Heremans, Harry Heimberg, John Casey, Kevin Docherty

**Affiliations:** 1 School of Medical Sciences, University of Aberdeen, Institute of Medical Sciences, Foresterhill, Aberdeen, AB25 2ZD, United Kingdom; 2 Department of Surgery, University of Edinburgh, Edinburgh Royal Infirmary, Edinburgh, EH16 4SU, United Kingdom; 3 Endocrinology Unit, University/BHF Centre for Cardiovascular Science, Queen’s Medical Research Institute, University of Edinburgh, Edinburgh, EH16 4TJ, United Kingdom; 4 Diabetes Research Centre, Vrije Universiteit Brussel, B1090 Brussels, Belgium; INSERM UMRS 1138, FRANCE

## Abstract

Transcription factor mediated lineage reprogramming of human pancreatic exocrine tissue could conceivably provide an unlimited supply of islets for transplantation in the treatment of diabetes. Exocrine tissue can be efficiently reprogrammed to islet-like cells using a cocktail of transcription factors: Pdx1, Ngn3, MafA and Pax4 in combination with growth factors. We show here that overexpression of exogenous Pax4 in combination with suppression of the endogenous transcription factor ARX considerably enhances the production of functional insulin-secreting β-like cells with concomitant suppression of α-cells. The efficiency was further increased by culture on laminin-coated plates in media containing low glucose concentrations. Immunocytochemistry revealed that reprogrammed cultures were composed of ~45% islet-like clusters comprising >80% monohormonal insulin^+^ cells. The resultant β-like cells expressed insulin protein levels at ~15–30% of that in adult human islets, efficiently processed proinsulin and packaged insulin into secretory granules, exhibited glucose responsive insulin secretion, and had an immediate and prolonged effect in normalising blood glucose levels upon transplantation into diabetic mice. We estimate that approximately 3 billion of these cells would have an immediate therapeutic effect following engraftment in type 1 diabetes patients and that one pancreas would provide sufficient tissue for numerous transplants.

## Introduction

Type 1 diabetes results from the autoimmune destruction of the insulin-producing pancreatic β-cells that are localised in islets of Langerhans. The most common form of treatment is the exogenous supply of insulin, which efficiently reduces blood glucose levels but is unable to mimic the tight glycaemic control provided by endogenous hormone production as there is no glucose-insulin feedback control. This may lead to the development of further complications, including life-threatening hypoglycaemia. The development of the Edmonton protocol represented a landmark in the treatment of type 1 diabetes, by establishing that transplantation of isolated cadaveric islets provides much superior glycaemic control and prolonged insulin independence [1]. The wide application of this cell therapy is, however, limited by the shortage of available donor islets. Thus, several strategies have been devised aimed at generating a replenishable supply of β-cells for transplantation. These include derivation of β-cells from pluripotent cells [2–11], and a variety of adult tissues that includes liver [12–15], and exocrine pancreas [16–33].

We have previously shown that human exocrine tissue that is left over from the islet isolation procedure can be reprogrammed towards insulin producing cells *ex vivo*, using a combination of the four pancreatic transcription factors (TFs) Pdx1, MafA, Ngn3 and Pax4 in combination with the small molecules betacellulin, exendin-4 and nicotinamide. Crucially, generation of β-like cells was dependent upon suppressing the epithelial to mesenchymal transition (EMT) that the exocrine tissue undergoes when placed in culture [34,35]. The resultant cell population secreted insulin in response to glucose at concentrations within the physiological range, and prevented the onset of diabetes when implanted in a diabetic mouse model [30].

In the present study we report the generation of mature, functional and clinically relevant β-cells from human exocrine pancreas. The major finding was that knockdown of endogenous ARX expression resulted in improved β-cell lineage specification. The resultant β-like cells exhibited many of the properties of fully differentiated adult β-cells, expressed insulin at therapeutically significant levels, processed insulin in a similar manner to adult β-cells, were glucose responsive, and contained insulin-secretory granules. Importantly, these β-like cells released insulin into the blood stream and were able to normalise blood glucose levels shortly after transplantation into diabetic mice.

## Materials and Methods

### Preparation of human exocrine pancreatic fractions

All human tissue was procured with written informed consent from the donor or next of kin and with ethical approval from the North of Scotland and Chelsea Research Ethics Committees (REC reference numbers 10/S0802/12 and 15/LO/2206). Human islets were isolated from brain-dead adult donor pancreata at the Scottish Islet Isolation Laboratory, Edinburgh, UK, under GMP conditions. For the five donors used in this study (3 males and 2 females) the mean donor age was 35.6 ± 8.5 years (range 23–44 years) and BMI 29.4 ± 4.4 kg/m^2^ (range 25.6–30.4 kg/m^2^). The left over, exocrine enriched fraction, was immediately transported to the reprogramming laboratory (typically within 8–10 h), where an aliquot was stained for insulin with dithizone. Islets present within the partially digested exocrine tissue represented less than 1% (0.9 ± 0.3%, n = 5 preparations) of the total tissue. This exocrine-enriched fraction was then cryopreserved in 90% foetal bovine serum (FBS, Gibco, Life Technologies, Paisley, UK) and 10% DMSO (Sigma Aldrich, Dorset, UK) at a density of 300,000 exocrine clusters per vial.

### Reprogramming human exocrine pancreatic fractions

**Fig 1:**
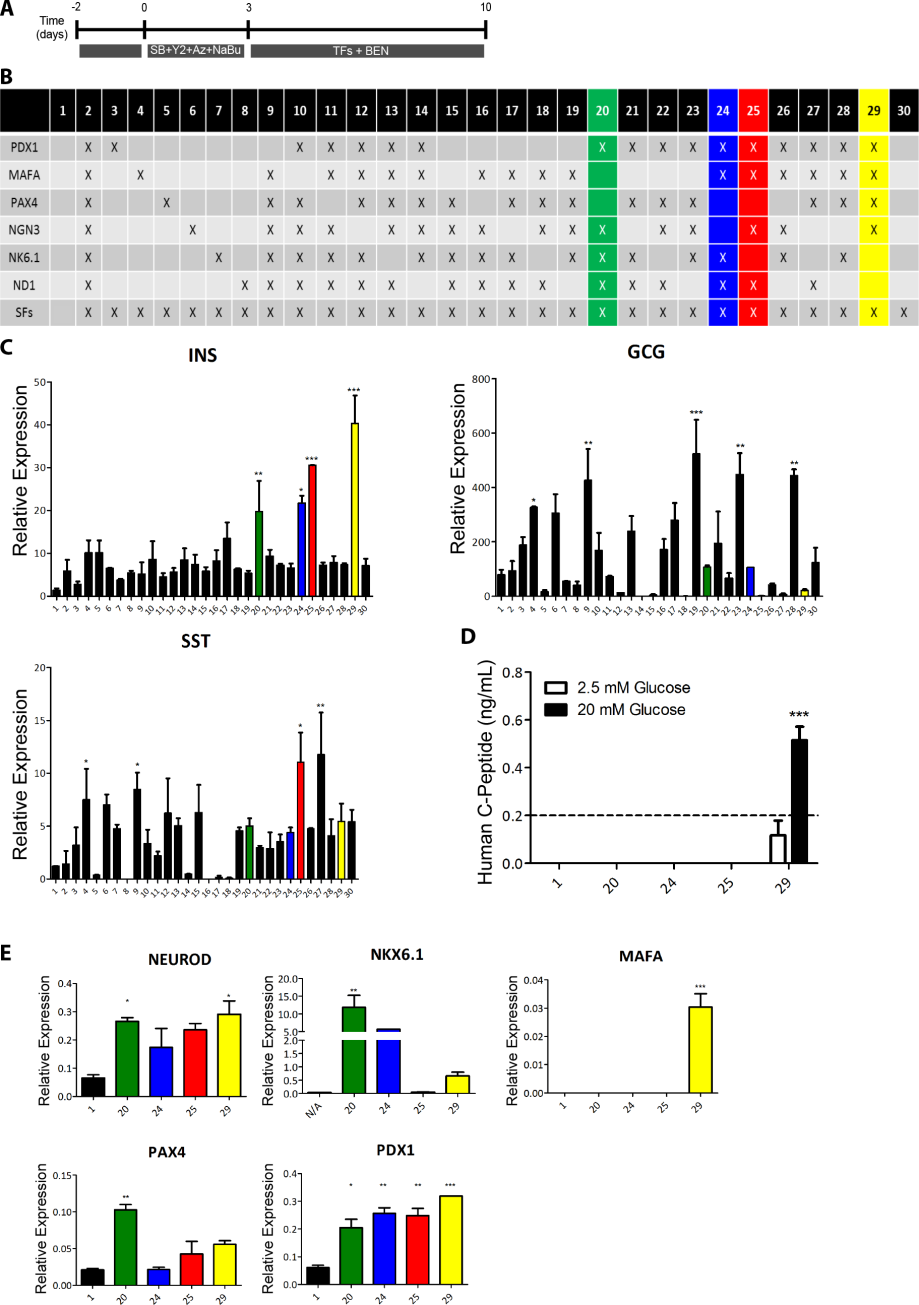
NeuroD and Nkx6.1 overexpression does not enhance reprogramming towards β-cells. **(A)** Schematic representation of the reprogramming protocol. **(B)** Schematic representation of the various transcription factor (TF) combinations analysed during the reprogramming protocol. **(C)** RT-qPCR analysis of the three main endocrine hormones insulin (INS), glucagon (GCG) and somatostatin (SST) after reprogramming with each TF combination. Expression was normalised to glyceraldehyde 3-phosphate dehydrogenase. **(D)** Release of human C-peptide to the culture medium by untreated (N/A) and reprogrammed cells treated with the TF combinations that induced higher insulin expression levels. The culture medium was harvested after 90 min incubation in 2.5 mM or 20 mM glucose. **(E)** RT-qPCR analysis of pancreatic endocrine transcription factors in untreated (N/A) or reprogrammed cells treated with the TF combinations that induced higher insulin expression levels. Expression was normalised to glyceraldehyde 3-phosphate dehydrogenase. Data are representative of triplicate experiments and are represented as mean + standard error of the mean. A one way ANOVA was performed followed by a Dunnet post hoc test to compare all treatment groups with the control group (N/A), where ***P < 0.001, ** *P* <0.01, *P < 0.05.

Exocrine tissue was plated at a density of 4000 clusters per well on a 6-well plate and cultured for two days in RPMI 1640 (Gibco, Life Technologies) supplemented with 10% foetal bovine serum (FBS), 10 mM HEPES and 1 mM sodium pyruvate (all from Gibco). After 48 h, the clusters adhered to the culture surface forming a monolayer of 1–2 x 10^5^ cells per well. These cells were subsequently cultured in serum free medium (SFM, RPMI 1640, 1% BSA (Sigma) and insulin-transferrin-selenium (Gibco)) supplemented with 1 μM 5-aza-2'-deoxycytidine, 1 mM sodium butyrate, 10 μM SB431542 and 2 μM Y27632 for 3 days. On day 4 the cells were transduced with replication-deficient adenoviruses encoding mouse Pdx1, Ngn3, MafA and Pax4, at a multiplicity of infection (MOI) of 25 each. The cells were then cultured in SFM supplemented with 1 nM betacellulin (Tocris, Bristol, UK), 10 nM exendin-4 (Sigma) and 10 mM nicotinamide (Sigma) for an additional 6 days, as previously described (Fig 1A and [30]). For screening of additional TFs replication-deficient adenoviruses encoding mouse NeuroD and/or Nkx6.1 were added at day 4, at an MOI of 25 each (Fig 1A and 1B).

### Preparation of adenoviruses

Recombinant adenoviruses encoding the mouse sequences of Pdx1, MafA, Ngn3, Pax4, NeuroD and Nkx6.1 [27] were prepared using the Ad-Easy system (Agilent Technologies, Edinburgh, UK). Transduction was performed in serum free RPMI for 4h at a multiplicity of infection (MOI) of 25 for each virus.

### Quantitative Reverse Transcription Polymerase Chain Reaction (RT-qPCR)

RT-qPCR was performed as previously described [30]. The TaqMan probes used are listed in S1 Table. Data were analysed using the 2^-ΔCT^ method [36].

### siRNA-based knockdown

**Fig 2:**
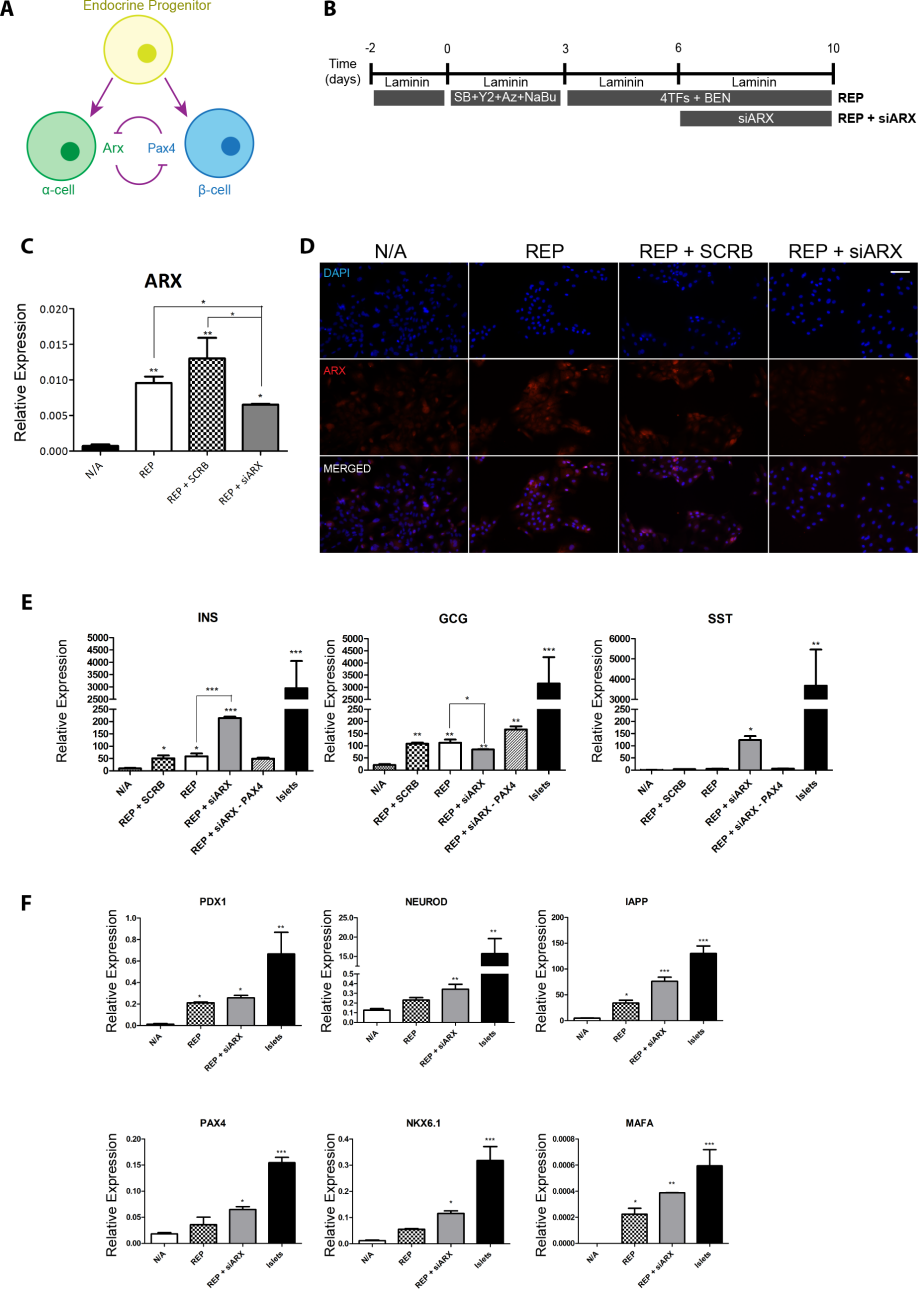
ARX knockdown by siRNA improves maturation of reprogrammed β-cells. **(A)** Schematic representation of the reprogramming protocol. The standard protocol (REP) consisted in plating the exocrine clusters in tissue culture dishes and allowing them to attach for 48h, followed by 3 day culture in serum free medium (SFM) with 10 μM SB431542 (SB), 2 μM Y27632 (Y2), 1 μM 5-Aza-2’deoxycytidine (Aza) and 1 mM sodium butyrate (NaBu). On day 4 cells were transduced with adenoviruses containing Pdx1, Ngn3, Pax4 and MafA, followed by 7 days in SFM with 1 nM betacellulin, 10 nM exendin-4 and 10 nM nicotinamide (BEN). The new protocol (REP + siARX) included the transfection with an siRNA targeting ARX at day 6 of the protocol. **(B)** Schematic representation of the interplay between the TFs PAX4 and ARX during the late stages of α- and β-cell development. **(C)** RT-qPCR analysis of endogenous ARX in untreated (N/A), REP cells, REP cells transfected with a scrambled siRNA (REP + SCRB) and REP + siARX cells. Expression was normalised to glyceraldehyde 3-phosphate dehydrogenase. Data are representative of triplicate experiments and represented as mean ± SEM. A one way ANOVA was performed followed by a Bonferroni post hoc test to compare all treatment groups, where ***P < 0.001, ** *P* <0.01, *P < 0.05. **(D)** Immunocytochemistry for ARX in untreated (N/A), REP cells, REP cells transfected with a scrambled siRNA (REP + SCRB) and REP + siARX cells. Scale bar = 50 μm. **(E)** RT-qPCR analysis of insulin, glucagon and somatostatin in untreated (N/A), REP cells, REP cells transfected with a scrambled siRNA (REP + SCRB), REP + siARX cells, REP + siARX in the absence of exogenous Pax4 (REP+siARX–PAX4) and in human islets. Expression was normalised to GAPDH. Data are representative of triplicate experiments. A one way ANOVA was performed followed by a Dunnet post hoc test, ***P < 0.001, ** *P* <0.01, *P < 0.05. **(F)** RT-qPCR analysis of late β-cell markers in untreated (N/A), REP cells, REP cells transfected with a scrambled siRNA (REP + SCRB), REP + siARX cells, REP + siARX in the absence of exogenous Pax4 (REP+siARX–PAX4) and in human islets. Expression was normalised to GAPDH. Data are representative of triplicate experiments. A one way ANOVA was performed followed by a Dunnet post hoc test, ***P < 0.001, ** *P* <0.01, *P < 0.05.

**Fig 3:**
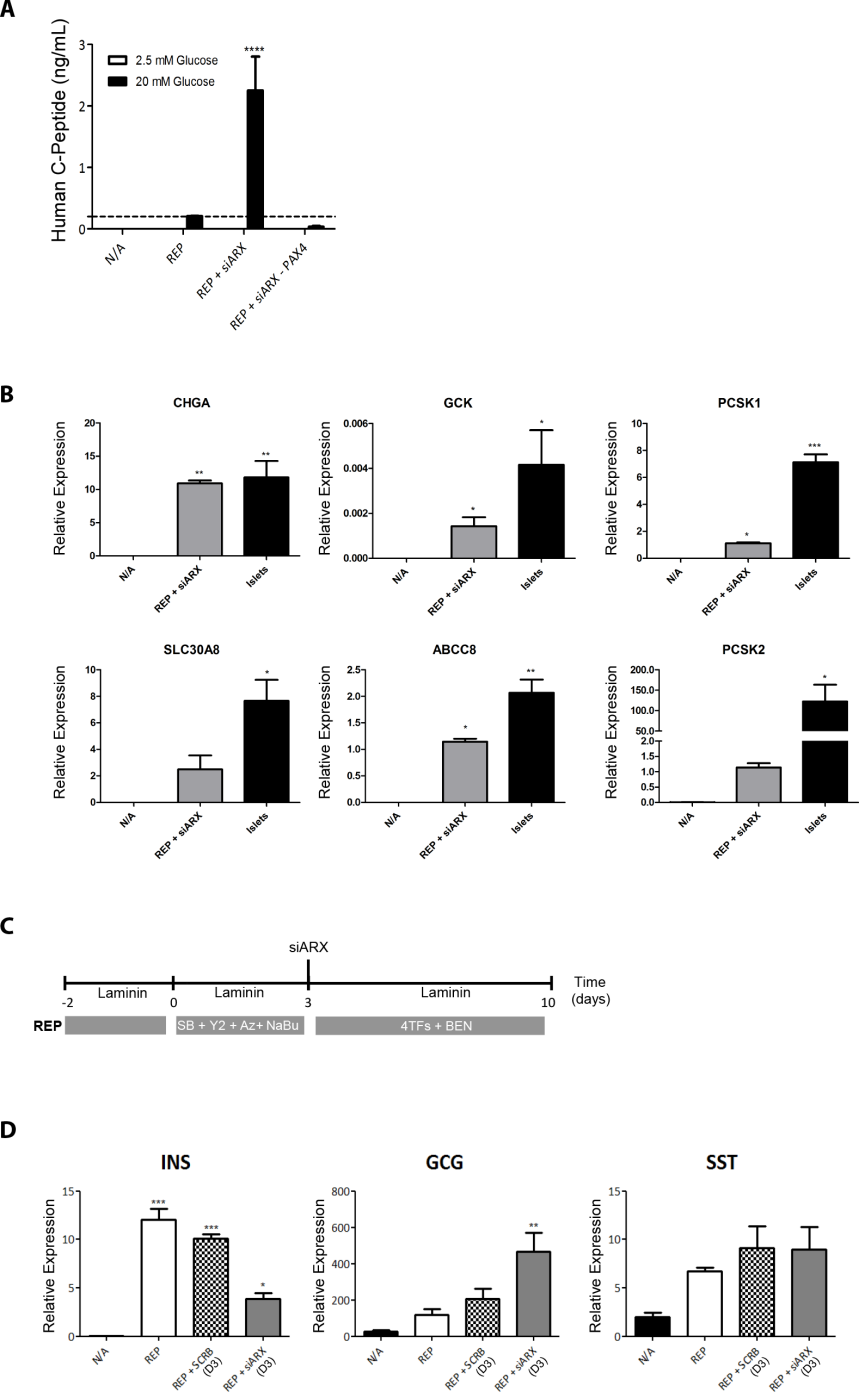
REP + siARX improves functionality of reprogrammed β-cells. **(A)** Release of human C-peptide in static incubation (90 min) in response to low (2.5 mM) and high (20 mM) glucose concentrations. A two way ANOVA was performed followed by a Bonferroni post hoc test, where ****P < 0.0001. **(B)** RT-qPCR analysis of the late functionality markers chromogranin A (CHGA), prohormone convertases (PCSK1 and PCSK2), glucokinase (GCK), K_ATP_ channel subunit SUR1 (SLC30A8) and zinc transporter (ABCC8) in untreated (N/A), REP+siARX cells and adult islets. Expression was normalised to GAPDH. Data are representative of triplicate experiments. A one way ANOVA was performed followed by a Dunnet post hoc test. Asterisks on top of bars represent comparisons with N/A, where *P < 0.05. **(C)** Schematic representation of the reprogramming protocol with inclusion of ARX knockdown by siRNA at the early stages (day 3) of the protocol. **(D)** RT-qPCR analysis of the three main endocrine hormones insulin (INS), glucagon (GCG) and somatostatin (SST) in untreated (N/A), REP cells, REP + SCRB (D3) or REP + siARX (D3). Expression was normalised to GAPDH. Data are representative of triplicate experiments. A one way ANOVA was performed followed by a Dunnet post hoc test to compare all treatment groups with the control group (N/A), where ***P < 0.001, ** *P* <0.01, *P < 0.05.

Knockdown of ARX in reprogrammed cells was performed by transfection in SFM with a pool of four specific targeting small inhibitory RNAs, or scrambled controls (Dharmacon, Loughborough, UK), using the transfection reagent Dharmafect 1 (Dharmacon), at days 6 (Fig 2A) or 3 (Fig 3C) of the protocol.

### Immunocytochemistry and immunohistochemistry

Immunostaining was performed as previously described [9], using the antibodies listed in S2 Table.

### Hormone content

Cells were washed in PBS and resuspended in 70 μl 0.1 M KH_2_PO_4_ (pH 7.8). Cells were lysed by three 30s freeze-thawing cycles. Supernatants were analysed using specific ELISAs for human insulin, C-peptide and proinsulin (all from Mercodia, Uppsala, Sweden) and glucagon (BD biosciences, Oxford, UK). Total protein was quantified using a protein assay (Biorad, Hemel Hempstead, UK).

### C-peptide release

Cells were washed in PBS followed by 90 min incubation in Krebs Ringer-bicarbonate Hepes (KRHB) buffer containing 0.1 mM glucose. The cells were subsequently incubated for 90 min with KRHB buffer containing the indicated glucose concentrations, and where indicated, 0.5 mM diazoxide (Sigma) or 0.2 mM tolbutamide (Sigma). Media were analysed with a specific ELISA for human C-peptide (Mercodia).

### Transmission electron microscopy

Cells were detached from plates using Accutase (BD Biosciences, Oxford, UK) and fixed in 2.5% glutaraldehyde in 0.1M sodium cacodylate buffer at 4°C overnight. Cells were post-fixed with 1% osmium tetroxide for 1 h followed by embedding in epoxy resin and dehydrated in a series of ethanol washes for 20 min, followed by embedding in epoxy resin, placing into moulds, and polymerised at 65°C for 48 h. Sections were taken between 75 and 90 nm on a Leica Ultracut E (Leica, Wetzlar, Germany) and placed on formvar/carbon grids. For immunogold labeling, cells were fixed in 0.2% glutaraldehyde and 2% paraformaldehyde. Sections were placed on nickel grids and stained using mouse anti-insulin (1:200, Sigma) and binding was identified using a goat anti-mouse protein A gold conjugate (1:40, 10 nm labels, Aurion, Wageningen, Netherlands). Images were observed on a JEOL JEM-1400 Plus TEM, and captured using an AMT UltraVue camera (Woburn, MA, USA).

### Animal studies

All animal experiments were performed under UK Home Office regulations (Project License 60/4242) and with the approval of the University of Aberdeen Animal Welfare and Ethical Review Body. Male 8–10 week old Scid/Beige mice (C.B.17/IcrHsd-Prkdc^scid^/Lyst^bg-J^) were obtained from Harlan Laboratories (Blackthorn, UK) and maintained on a 12 h light/dark cycle with *ad libitum* access to a standard irradiated diet (Harlan Laboratories). Mice were fasted for 4 h before rendered diabetic by three intraperitoneal injections of 75 mg/kg streptozotocin (STZ) on consecutive days. Five million cells were grafted under the left kidney capsule as previously described [9]. A glucose tolerance test was performed following an intraperitoneal injection of 2 mg/kg D-glucose (after 4h fasting). All animals subjected to a subcapsular kidney transplant and nephrectomy procedures were anesthetized with a mixture of isofluorane and oxygen. Analgesia (0.1mk/kg Buprenorphine) was used before and following the procedures to minimise pain. Body temperature was kept at 37°C throughout the procedure to minimise discomfort. At the end of the experiment all animals were sacrificed by cervical dislocation.

## Results

### Transcription factor-mediated reprogramming of pancreatic exocrine tissue

We have previously shown that the human exocrine tissue, obtained as a by-product of the islet isolation procedure, can be reprogrammed towards insulin producing β-like cells [30]. The exocrine tissue is plated on tissue culture dishes for 48h to form a monolayer. The cells then undergo an epithelial to mesenchymal transition (EMT) [37] over a period of days with rapid loss of insulin (endocrine) and amylase (acinar). Interestingly, as previously reported [25], the acinar cells dedifferentiate via an intermediate that co-expresses amylase and CK19. Our previous in vitro genetic lineage tracing studies confirmed that the few residual β-cells and acinar cells contribute to the resultant mesenchymal stromal cell (MSC) population [30]. This MSC population expresses characteristic cell surface markers, can be differentiated towards osteogenic, chondrogenic and adipogenic lineages, and repeatedly passaged. We reported previously [30] that efficient reprogramming towards β-cells was dependent on inhibiting EMT using the TGFβ inhibitor SB431542 (SB) and the Rho kinase (ROCK) inhibitor Y27632 (Y2). Sodium butyrate (NaBu) and Aza-2’deoxycytidine (Aza) were also included to modulate the chromatin structure (Fig 1A). A detailed time course analysis showed that there was no detectable insulin by RT/qPCR (S1A Fig) or immunocytochemistry (S1B Fig) when the cells were cultured for 72 h in the presence of these reagents, i.e. they did not induce selective retention and proliferation of β-cells. After 72h the cells were transduced with adenoviruses containing Pdx1, MafA, Ngn3 and Pax4 (4TFs) and cultured for 7 days in media supplemented with betacellulin, exendin-4 and nicotinamide (BEN, Fig 1A). Although exhibiting many of the properties of fully differentiated β-cells, the resultant cells expressed insulin at only 1% of that in adult human islets. The present studies aimed at improving the protocol in order to obtain cells that more closely resembled mature β-cells.

We first investigated whether addition of two other pancreatic (TFs), Nkx6.1 and NeuroD, would further drive reprogramming towards a more mature β-cell phenotype. These two TFs, were overexpressed individually or in combination, through recombination deficient adenoviruses along with the original cocktail of Pdx1, MafA, Ngn3 and Pax4 (combination 29, Fig 1A and 1B). No significant increase in insulin expression was observed upon addition of either Nkx6.1 or NeuroD to the original cocktail (combinations 13 and 14, Fig 1C). Futhermore, the original cocktail of Pdx1, Ngn3, MafA and Pax4 [30] was the most efficient in inducing insulin expression (condition 29, Fig 1B and 1C).

Combinations 20, 24, 25 and 29 were selected for further analysis based on their ability to induce high insulin mRNA levels (Fig 1C). Interestingly, the original TF combination 29 was the only able to generate glucose responsive β-like cells (Fig 1D) and the only to induce expression of the β-cell maturation factor MAFA, indicating that endogenous MAFA expression might be necessary for the functionality of the reprogrammed β-cells, and that MAFA activation might be dependent on exogenous Pax4 overexpression, since this TF was absent from conditions 20, 24 and 25.

Overall, these results suggest that the most efficient TF combination for the *ex vivo* reprogramming of exocrine pancreatic cells towards β-cells results from the concerted actions of Pdx1, Ngn3, MafA and Pax4. Inclusion of Pax4 appears to be crucial for generating glucose responsive beta-like cells, as reported previously [41].

### Knock-down of endogenous ARX enhances insulin production

In the mouse, during the late stages of pancreatic development, the interplay between the TFs Arx and Pax4 determines the establishment of either the α- or β-cell lineages (Fig 2A) [38]. Moreover, Arx inactivation plays an important role in maintaining β-cell identity in mouse and human islets [39,40]. Given the crucial role of Pax4 for the functionality of the reprogrammed β-cells [30,41], we hypothesised that transient knockdown of endogenous ARX would promote further maturation during reprogramming. These experiments were performed on laminin-coated plates [42,43] in low glucose (2.5 mM) RPMI [44], which had a moderate but significant effect on insulin mRNA levels (S2A and S2B Fig). It should be noted that the antioxidant vitamin C and the vitamin E analogue 6 hydroxy 2,5,7,8 tetramethaylchroman 2 carboxylic acid (Trolox), which can aid in the reprograming of somatic cells towards pluripotency [45,46], as well as the thyroid hormone 3,5,3-triiodo-L-thyronine (T3), which has been implicated in the maturation of the β-cell lineage [47] and in the reprogramming of exocrine pancreatic cells towards insulin producing cells [48,49], had no effect on insulin mRNA levels (S2C Fig).

A pool of four short interfering RNAs directed against ARX was added at day 6 (Fig 2B), i.e. 2 days after addition of the 4 TFs. Under these conditions endogenous ARX mRNA levels decreased by 50% (Fig 2C and 2D). Reprogramming (REP) in the absence of siARX (Fig 2E) resulted in insulin mRNA levels of 59.26 ± 11.25 (arbitrary units) and was unaffected by including a control scrambled siRNA. Reprogramming in the presence of siARX (REP+siARX) resulted in a significant increase in insulin mRNA levels to 214.30 ± 5.57 (Fig 2E), which was reproducible between exocrine cells from four additional donors (S2D Fig). The effect of siARX was dependent on the presence of Pax4 in the reprogramming cocktail (Fig 2E). Expression of additional key β-cell markers was also increased in REP+siARX as compared to REP (Fig 2F). Inclusion of siARX significantly decreased glucagon mRNA and protein levels (Fig 2E and S2E and S2F Fig); however in the absence of Pax4 glucagon mRNA levels increased, thus emphasising the key inhibitory effect of Pax4 on glucagon expression. While somatostatin mRNA levels increased significantly by inclusion of siARX (Fig 2E), this was entirely due to somatostatin expression in very few isolated cells as shown by immunocytochemistry (S3B Fig).

Glucose sensitivity was markedly enhanced in REP+siARX versus REP, and this effect was dependent on the presence of exogenous Pax4 (Fig 3A), which supports our previous observation that Pax4 overexpression is important not only for inducing insulin expression, but also for the functionality of the reprogrammed insulin-producing cells [41]. In keeping with this observation, REP+siARX cells expressed mature endocrine markers at levels close to those of adult islets (Fig 3B). Finally, the timing of endogenous ARX knockdown was crucial. Whereas knockdown at the later stages of the protocol (Fig 2A) resulted in a significant increase in insulin expression (Fig 2E), the opposite was observed when ARX was inhibited earlier in the protocol (Fig 3C and 3D). This is in keeping with studies on the differentiation of hESCs, containing a deletion in the ARX gene, which showed a temporal effect of exogenous ARX re-expression on the resulting endocrine cell population [50].

### REP+siARX generates functional β-like cells

**Fig 4:**
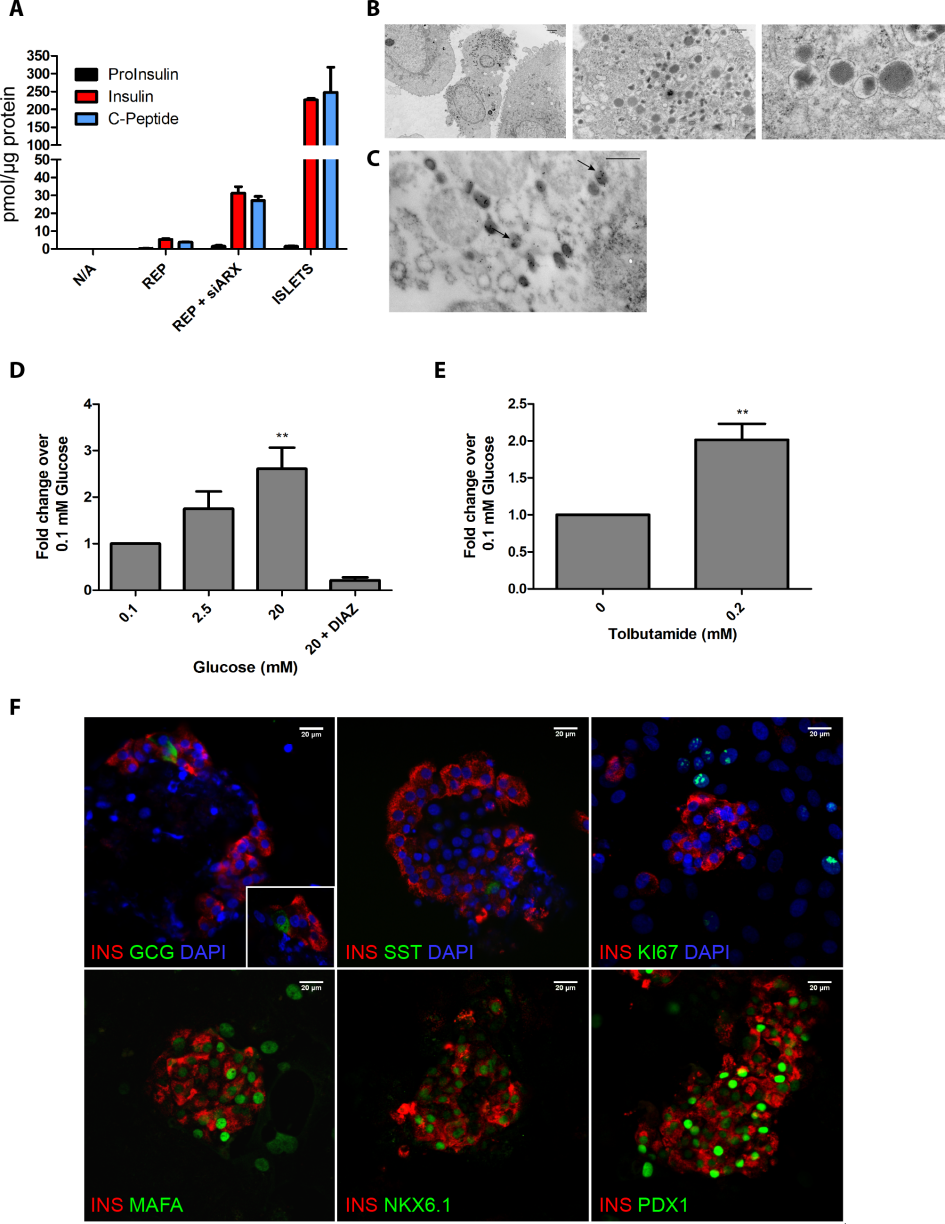
REP + siARX closely resemble adult β-cells. **(A)** Human proinsulin, insulin and C-peptide content of untreated (N/A), REP+siARX, REP cells and human islets, normalised to total protein content. **(B)** Electron microscopy of REP + siARX cells. Scale bar = 2 μm (top left), 0.5 μm (right) and 0.1 μm (bottom right). **(C)** Immunogold labelling of insulin granules in REP + siARX cells. Scale bar = 0.5 μm. **(D-E)** Human C-peptide release under different secretory conditions. A one way ANOVA was performed followed by a Dunnet post hoc test, **P < 0.01. **(F)** Immunocytochemistry of REP+siARX cells. Scale bar = 20 μm.

Equivalent proportions of C-peptide and insulin, and very low levels of proinsulin were detected in reprogrammed cells (REP+siARX) and adult human islets, suggesting that proinsulin was efficiently processed to insulin in the reprogrammed cells (Fig 4A). The insulin content of REP+siARX cells was 14.8±3.2% of the levels present in adult human islets, while REP produced only 2.4±0.6% of the islet levels (Fig 4A). Electron microscopy confirmed that the newly reprogrammed REP+siARX β-cells were capable of storing insulin in dense-core granules (Fig 4B and 4C) with a morphology typical of mature β-cells (S3A Fig). High glucose in the culture medium stimulated the secretion of insulin from REP+siARX cells via mechanisms that are similar to those in adult human islets; i.e. in a manner that is inhibited in the presence of the K_ATP_ channel activator diazoxide (Fig 4D) and stimulated by the K_ATP_ channel blocker tolbutamide (Fig 4E).

Immunocytochemistry showed that REP+siARX cells aggregated in structures with similar morphology to adult islets, surrounded by MSCs (Fig 4F and S3C Fig). In the reprogrammed cultures, ~40% of the cells produced C-peptide, while less than 5% of the cells were positive for glucagon and a very small percentage (<2%) expressed somatostatin (S3B Fig). The small numbers of somatostatin and glucagon producing cells were found to be monohormonal (Fig 4F). The reprogrammed β-like cells in these clusters were positive for the mature β-cell TFs MAFA, NKX6.1 and PDX1 (Fig 4F) and did not co-stain with the exocrine markers amylase or CK19 (S3C Fig). Unlike non-reprogrammed cells (S3D Fig), the reprogrammed β-cells did not stain positive for the mesenchymal marker vimentin (S3C Fig) or the proliferation marker Ki67 (Fig 4F), in accordance with the characteristics of adult β-cells.

In summary, these newly reprogrammed cells closely resemble adult β-cells, as they are able to efficiently store and process insulin, secrete insulin in response to a rise in glucose concentrations and contain a large number of the phenotypic characteristics of adult β-cells.

### REP+siARX β-cells reverse diabetes in streptozotocin-treated mice

**Fig 5:**
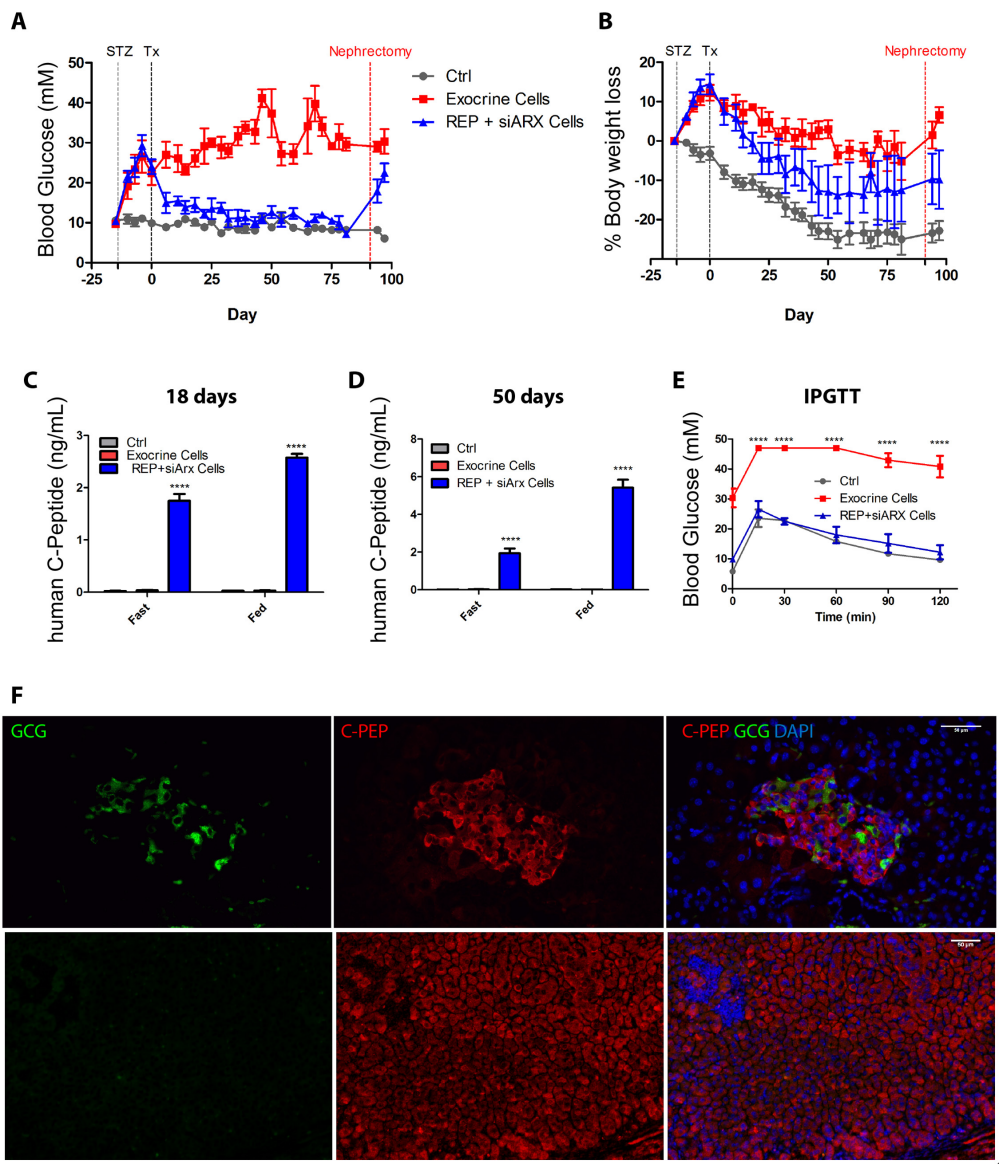
REP + siARX cells rescue diabetes in Streptozotocin-treated mice. Blood glucose levels **(A),** percentage of body weight loss **(B)**, serum human C-peptide levels **(C-D)**, and intraperitoneal glucose tolerance test **(E)** of diabetic mice grafted with 5 X 10^6^ REP+siARX cells (n = 5), non-reprogrammed exocrine cells (n = 6) and control non diabetic and non-grafted mice (n = 6). STZ—streptozotocin administration; Tx—time of surgery; Nephrectomy—time of graft removal. Serum samples were collected after 4h starvation (Fast) or *ad libitum* feeding (Fed) for C-peptide detection. A two way ANOVA was performed followed by a Bonferroni post hoc test, ****P < 0.0001. **(F)** Immunostaining for C-peptide and glucagon in REP+siARX grafts. Nuclei were counterstained with DAPI. Scale bar = 50 μm.

To evaluate their *in vivo* function, the reprogrammed (REP+siARX) β-like cells were transplanted under the kidney capsule of immunocompromised Scid/Beige mice, rendered hyperglycaemic by three consecutive doses of streptozotocin (STZ). The reprogrammed cells rescued diabetes shortly after engraftment, reducing blood glucose to normal physiological levels (Fig 5A) and stabilising body weight (Fig 5B). Human C-peptide was detected in the serum of mice transplanted with REP+siARX cells after 18 days, the earliest time point after surgery when this assay could be performed (Fig 5C). Serum human C-peptide levels increased by ~2 fold after 50 days (Fig 5D). Importantly the levels of circulating human C-peptide of ~6 ng/ml indicate improved functionality when compared with the values observed (0.6 ng/ml) when equivalent numbers of REP cells were grafted into streptozotocin-diabetic NOD/Scid mice in our previous study [30]. Mice transplanted with non-reprogrammed exocrine cells remained diabetic throughout the course of the experiment and no human C-peptide could be detected in their blood (Fig 5A–5D). An intraperitoneal glucose tolerance test revealed that the reprogrammed β-cells were functionally similar to endogenous β-cells (Fig 5E). Removal of the transplanted kidney at the end of the study confirmed that diabetes rescue was due to the presence of reprogrammed cells, and immunohistochemistry revealed the presence of high numbers of monohormonal insulin-producing cells and a small number of glucagon-producing cells within the grafts, in islet-like structures (Fig 5F).

In conclusion, the present study has shown that inhibition of ARX plays an important role in *ex vivo* reprogramming of exocrine pancreatic cells towards functional β-cells. The reprogrammed cells resemble mature β-cells in that that are capable of producing significant amounts of insulin (33.5±7.3 pmol/μg protein) when compared to human islets (226.7±9.5 pmol/μg protein), are glucose responsive, phenotypically stable, and are capable of rescuing diabetes in an *in vivo* setting.

## Discussion

Over 95% of the exocrine pancreas is discarded following each islet isolation procedure. The ability to reprogramme this tissue towards functional β-cells could circumvent the need for multiple donor pancreata to treat a single patient and would allow one pancreas to treat several patients. In fact we have previously shown that the discarded exocrine material could be reprogrammed towards β-like cells. However, these cells produced insulin levels well below those found in adult human islets [30]. Here we report the *ex vivo* generation from human exocrine pancreas of mature functional, i.e. glucose responsive, β-cells that contain insulin at levels around 15–30% of those in human islets. These cells would have an immediate therapeutic effect if implanted in patients with type 1 diabetes. We know this from the fact that in type 1 diabetes there is a progressive loss of β-cells that occurs over a number of years before the onset of symptoms, such that at clinical presentation most patients have residual (~10%) β-cell function [37]. This suggests that reinstating a fraction of the normal β-cell mass would have a therapeutic effect. Moreover, successful transplants can be achieved with donor islets equivalent to as little as 30–40% of the total islet content of a pancreas.

The reprogrammed β-like cells were monohormonal and formed islet-like structures with few glucagon- and somatostatin-expressing cells. The reprogrammed cells efficiently processed proinsulin to insulin, contained insulin storage granules, secreted insulin in response to glucose, and rescued diabetes in a streptozotocin mouse model. The diabetic mouse studies confirmed that the cells were phenotypically stable. The protocol was crucially dependent on overexpressing exogenous Pax4 and, at a later stage inhibiting endogenous ARX. The antagonistic roles of Arx and Pax4 have been well documented in pancreatic development in mice. Thus, Pax4 inhibits Arx expression in β-cells, while the reverse occurs for α-cell differentiation. Moreover, the Arx promoter is hypermethylated in adult β-cells [39] and Arx overexpression results in conversion of adult human β-cells towards α-cells [51]. We have previously shown that Pax4 plays a crucial role in generating functional β-cells [30,41], but the exact mechanisms are unclear.

With regard to the mechanisms, we have previously shown that successful TF-mediated conversion of pancreatic exocrine cells to β-like cells is dependent on suppressing, via inhibition of Rho Kinase and TGFβ1 signalling, the EMT that pancreatic tissue undergoes when placed in monolayer culture [30]. EMT is a multi-stage process [52] and it is likely that our protocol involves delaying transit through an early stage. During this early stage, amylase-positive cells disappear through an intermediate that co-expresses amylase and the ductal marker CK19 [25,30]. Further mechanistic studies at the single cell level will be required to ascertain whether this acinar/ductal hybrid cell is the source of the resultant β-like cells. In vivo studies in mice have shown that exogenous Pdx1, MafA and Ngn3 can induce the appearance of new β-cells within the pancreas. Furthermore, genetic lineage tracing studies demonstrated that these newly formed isolated β-cells likely arose through transdifferentiation of acinar cells [24]. It will be interesting to know if this transdifferentiation also occurred via an acinar/ductal intermediate.

**Fig 6:**
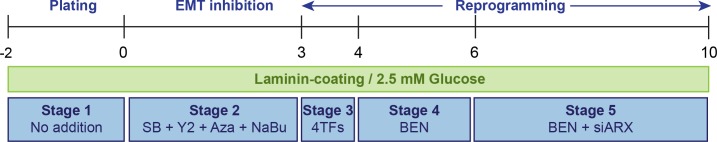
Final reprogramming protocol. Schematic representation of the final reprogramming protocol. The cells are plated in laminin-coated dishes for 48h (Stage 1). The cells are then cultured in serum free RPMI medium (SFM) containing 2.5 mM glucose. For the first 3 days (Stage 2), EMT is inhibited using Rho kinase (2 μM Y27632) and TGF-β (10 μM SB431542) inhibitors in combination with methyltransferase (1 μM 5’-Aza-2’-deoxycytidine) and histone deacetylase inhibitors (1 mM sodium butyrate). This is followed by transduction with the exogenous TFs (Stage 3) Pdx1, Ngn3, Pax4 and MafA and culture in SFM containing 1 nM betacellulin, 10 mM nicotinamide and 10 nM exendin-4. Inhibition of endogenous ARX by siRNA is performed at day 6 (Stage 4) and the cells are then maintained in SFM containing BEN until day 10 (Stage 5).

The final protocol (Fig 6) takes around 12 days and involves plating the isolated human exocrine tissue on laminin and culturing in media containing 2.5 mM glucose. Initially, the EMT is inhibited using Rho kinase and TGF-β inhibitors in combination with methyltransferase and histone deacetylase inhibitors. This is followed by transduction with the exogenous TFs Pdx1, Ngn3, Pax4 and MafA in media containing betacellulin, nicotinamide and exendin-4. Inhibition of endogenous ARX by siRNA is performed towards the final stages of the protocol. The final product is around 40% endocrine; the remaining cells are pancreatic MSCs which have added advantage in terms of the function of the transplanted tissue [53–55]. The cells are stable as evidenced by the prolonged appearance of human C-peptide in the blood of transplanted mice and the morphology of the transplanted aggregates at periods up to 100 days after transplantation.

In terms of the clinical applications of this protocol, there are at least five important criteria that would need to be met; 1. Do the cells express fully processed insulin at therapeutic levels? 2. Do the cells function, i.e. efficiently store, process and secrete insulin in response to glucose and other nutrients, in a manner similar to adult human islets? 3. Do the cells form aggregates that look like adult human islets? 4. Do the cells normalise blood glucose levels in an appropriate diabetic animal model? 5. Are the cells phenotypically stable? As described above our protocol meets all five of these criteria. It would therefore be interesting to compare this protocol with other approaches to obtaining an alternative supply of islets for transplantation. Such a goal, however, is hindered by the lack of quantitative data in most of the relevant studies. Many studies to date have relied heavily on RT-qPCR, immunocytochemistry and flow cytometry. Where the quantitative data are available our reprogrammed cells that make 33.5 pmol insulin/μg protein compare well with the functionality of human pluripotent cell (hPC)- derived islet progenitors β-cells [5], with the insulin content and functionality of hPC-derived fully differentiated β-like cells [10,11], and with β-like cells generated from human ductal-enriched cells [28].

In summary, our protocol generates β-cells that share many of the properties of adult endogenous β-cells and compare well with surrogate β-cells generated from hPCs. Our approach, however, has the advantage that the cells are not at any stage pluripotent, which has important safety considerations. In addition, our protocol is relatively simple, cost effective, adaptable to clinical grade good manufacturing (GMP) conditions, and at 12 days is significantly shorter than the time required to generate fully functional β-like cells from hPCs. We estimate that around 3–5 x 10^8^ reprogrammed cells would have a therapeutic effect if transplanted in diabetic patients; thus one donor pancreas could provide numerous (~10–12) islet grafts.

## Supporting Information

S1 Fig Delaying EMT does not preserve contaminating β-cells in culture. Expression of endocrine markers present in the initial exocrine tissue (-48h) is quickly lost after 48h in culture. EMT inhibition with SB, Y2, Aza and NaBu does not lead to re-expression of any of the endocrine hormones. At the same time, amylase expression is lost while the treatment results in the increase of the ductal marker CK19 and E-cadherin, supporting our previous observation of an existing intermediate amylase^+^/CK19^**+**^ cell population. **(A)** RT-qPCR analysis of endocrine and exocrine pancreatic markers at time points during the first five days of the reprogramming protocol. Expression was normalised to GAPDH. Data are representative of triplicate experiments. A one way ANOVA was performed followed by a Dunnet post hoc test, where ***P < 0.001, ** *P* <0.01 and *P < 0.05. **(B)** Immunocytochemistry for C-peptide and the exocrine marker CK19 during the first 72h of the reprogramming protocol. Nuclei were counterstained with 4',6-Diamidino-2-Phenylindole, Dihydrochloride (DAPI). Scale bar = 100 μm.
(TIF)

S2 Fig Laminin, low glucose and siARX favour β-cell reprogramming. **(A)** Laminin is the extracellular matrix component most favourable to endocrine reprogramming, resulting in increased expression levels of the three main pancreatic hormones insulin (INS), glucose (GCG) and somatostatin (SST). RT-qPCR analysis of insulin, glucagon and somatostatin in reprogrammed cells (REP) cultured in standard tissue culture dishes (CTRL) or dishes coated with extracellular matrix components, normalised to GAPDH. A two way ANOVA was performed followed by a Bonferroni post hoc test, ***P < 0.001, ** *P* <0.01, *P < 0.05. **(B)** Low glucose concentrations favoured insulin expression under reprogramming conditions, in contrast to higher glucose concentrations. RT-qPCR analysis of insulin, glucagon and somatostatin in N/A and REP cultured in various glucose concentrations, normalised to GAPDH. Data are representative of triplicate experiments. A two way ANOVA was performed followed by a Bonferroni post hoc test, ***P < 0.001, ** *P* <0.01, *P < 0.05. **(C)** Antioxidant compounds and thyroid hormone 3,5,3-triiodo-L-thyronine (T3) do not enhnance reprogramming towards the beta-cell lineage. RT-qPCR analysis of insulin, glucagon and somatostatin in N/A and REP cultured in the presence of several combinations of the antioxidants Vitamin C (10 μg/mL, Vit. C), Trolox (5 μM) and the thyroid hormone T3 (1 μM). Data are normalised to GAPDH and representative of triplicate experiments. A two way ANOVA was performed followed by a Bonferroni post hoc test, ***P < 0.001, ** *P* <0.01, *P < 0.05. **(D)** The reprogramming protocol is highly reproducible between exocrine preparations of distinct donors. RT-qPCR analysis of insulin following REP + siARX reprogramming in exocrine preparations from four distinct donors. **(E)** Inhibition of Arx decreases reprogramming towards glucagon-producing cells. Human glucagon content of untreated (N/A), REP, REP+siARX cells and human islets, normalised to total protein. **(F)** Immunostaining for glucagon and C-Peptide on REP and REP + siARX cells. Nuclei were counterstained with 4',6-Diamidino-2-Phenylindole, Dihydrochloride (DAPI). Scale bar = 20 μm.
(TIF)

S3 Fig REP+siARX mainly generates insulin producing cells. **(A)** Immunogold labelling of insulin granules in adult human islets. Scale bar = 0.05 μm. **(B)** Reprogramming generates 40% beta-like cells and less than 5% of other endocrine cell types. Immunocytochemistry for the three main pancreatic hormones after REP+siARX reprogramming. Nuclei were counterstained with 4',6-Diamidino-2-Phenylindole, Dihydrochloride (DAPI). Scale bar = 50 μm. Immunohistochemical quantification for each endocrine cell type. Data is represented as mean + standard error of the mean, where *** *P* <0.01. Five hundred cells were quantified from 5 different random field views for each replicate. **(C)** Reprogrammed cells do not co-express exocrine or mesenchymal markers. Immunocytochemistry in REP+siARX cells for insulin or C-peptide and the exocrine markers CK19 and amylase, and the mesenchymal marker vimentin. Nuclei were counterstained with 4',6-Diamidino-2-Phenylindole, Dihydrochloride (DAPI). Scale bar = 20 μm. **(D)** Non-reprogrammed cells dedifferentiate towards mesenchymal stromal cells and proliferate in culture. Immunocytochemistry for vimentin, C-peptide and ki67 of untreated exocrine cultures. Nuclei were counterstained with 4',6-Diamidino-2-Phenylindole, Dihydrochloride (DAPI). Scale bar = 20 μm.
(TIF)

S1 Table List of TaqMan probes used for RT-qPCR analysis. (DOCX)

S2 Table List of primary antibodies used for immunocytochemistry. (DOCX)
