# Supplementary figures and images for: Generation of Functional Beta-Like Cells from Human Exocrine Pancreas
Source: PLoS One. 2016 May 31;11(5):e0156204. doi: 10.1371/journal.pone.0156204 (PMC4887015; doi:10.1371/journal.pone.0156204)

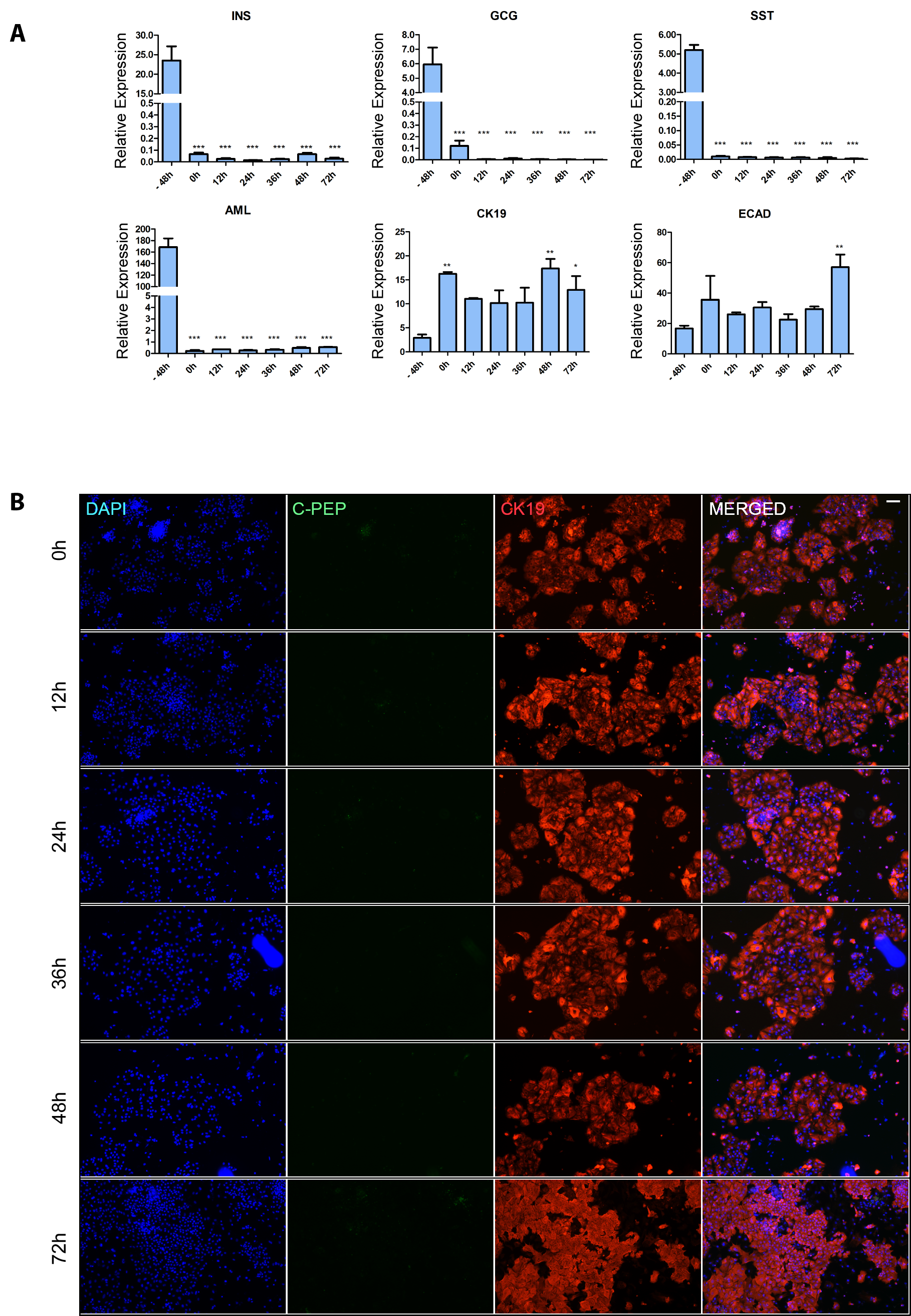

Supplement: S1 Fig — Expression of endocrine markers present in the initial exocrine tissue (-48h) is quickly lost after 48h in culture. EMT inhibition with SB, Y2, Aza and NaBu does not lead to re-expression of any of the endocrine hormones. At the same time, amylase expression is lost while the treatment results in the increase of the ductal marker CK19 and E-cadherin, supporting our previous observation of an existing intermediate amylase+/CK19+ cell population. (A) RT-qPCR analysis of endocrine and exocrine pancreatic markers at time points during the first five days of the reprogramming protocol. Expression was normalised to GAPDH. Data are representative of triplicate experiments. A one way ANOVA was performed followed by a Dunnet post hoc test, where ***P < 0.001, ** P <0.01 and *P < 0.05. (B) Immunocytochemistry for C-peptide and the exocrine marker CK19 during the first 72h of the reprogramming protocol. Nuclei were counterstained with 4',6-Diamidino-2-Phenylindole, Dihydrochloride (DAPI). Scale bar = 100 μm. (TIF) [file pone.0156204.s001.tif]

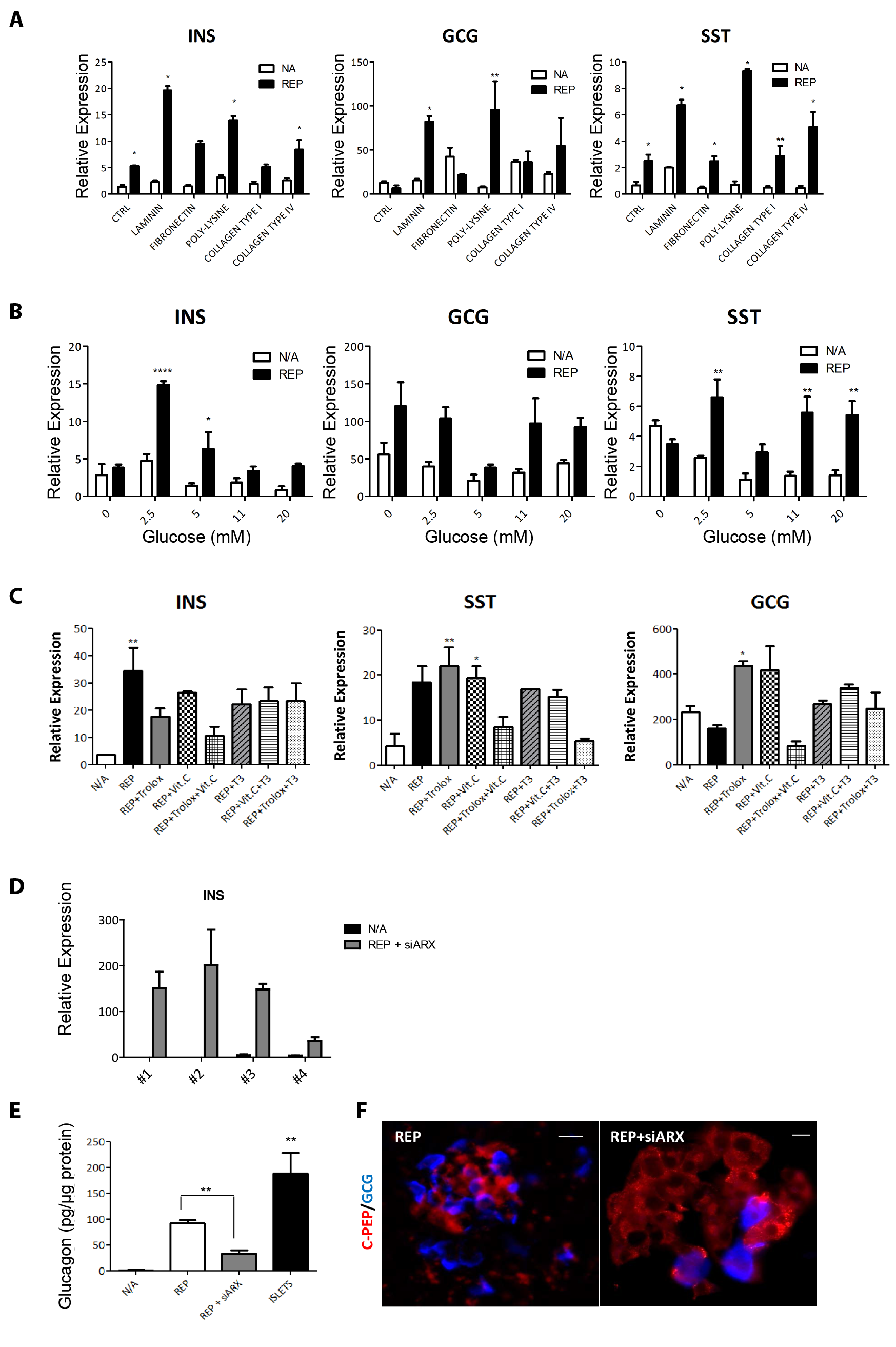

Supplement: S2 Fig — (A) Laminin is the extracellular matrix component most favourable to endocrine reprogramming, resulting in increased expression levels of the three main pancreatic hormones insulin (INS), glucose (GCG) and somatostatin (SST). RT-qPCR analysis of insulin, glucagon and somatostatin in reprogrammed cells (REP) cultured in standard tissue culture dishes (CTRL) or dishes coated with extracellular matrix components, normalised to GAPDH. A two way ANOVA was performed followed by a Bonferroni post hoc test, ***P < 0.001, ** P <0.01, *P < 0.05. (B) Low glucose concentrations favoured insulin expression under reprogramming conditions, in contrast to higher glucose concentrations. RT-qPCR analysis of insulin, glucagon and somatostatin in N/A and REP cultured in various glucose concentrations, normalised to GAPDH. Data are representative of triplicate experiments. A two way ANOVA was performed followed by a Bonferroni post hoc test, ***P < 0.001, ** P <0.01, *P < 0.05. (C) Antioxidant compounds and thyroid hormone 3,5,3-triiodo-L-thyronine (T3) do not enhnance reprogramming towards the beta-cell lineage. RT-qPCR analysis of insulin, glucagon and somatostatin in N/A and REP cultured in the presence of several combinations of the antioxidants Vitamin C (10 μg/mL, Vit. C), Trolox (5 μM) and the thyroid hormone T3 (1 μM). Data are normalised to GAPDH and representative of triplicate experiments. A two way ANOVA was performed followed by a Bonferroni post hoc test, ***P < 0.001, ** P <0.01, *P < 0.05. (D) The reprogramming protocol is highly reproducible between exocrine preparations of distinct donors. RT-qPCR analysis of insulin following REP + siARX reprogramming in exocrine preparations from four distinct donors. (E) Inhibition of Arx decreases reprogramming towards glucagon-producing cells. Human glucagon content of untreated (N/A), REP, REP+siARX cells and human islets, normalised to total protein. (F) Immunostaining for glucagon and C-Peptide on REP and REP + siARX cells. Nuc [file pone.0156204.s002.tif]

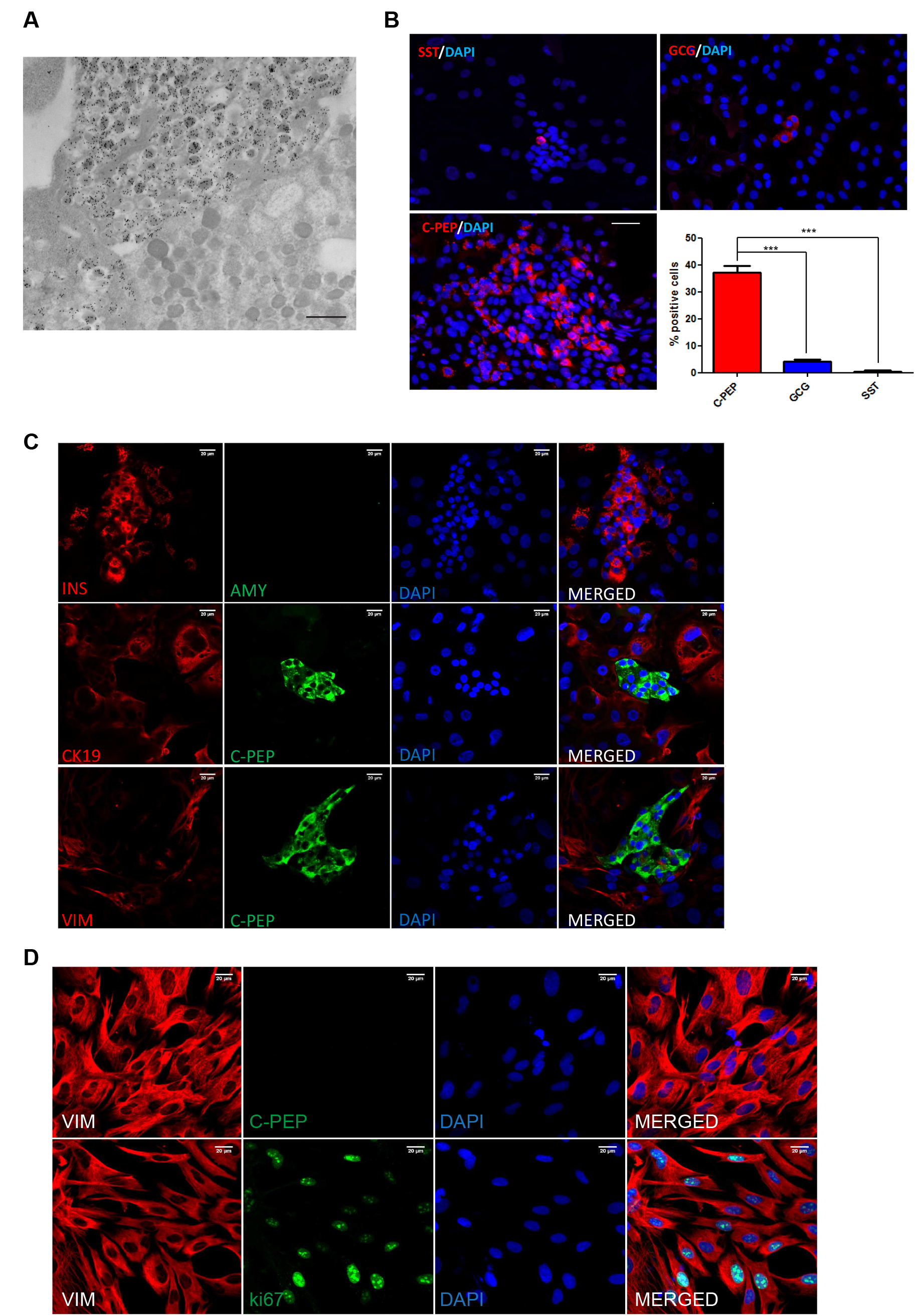

Supplement: S3 Fig — (A) Immunogold labelling of insulin granules in adult human islets. Scale bar = 0.05 μm. (B) Reprogramming generates 40% beta-like cells and less than 5% of other endocrine cell types. Immunocytochemistry for the three main pancreatic hormones after REP+siARX reprogramming. Nuclei were counterstained with 4',6-Diamidino-2-Phenylindole, Dihydrochloride (DAPI). Scale bar = 50 μm. Immunohistochemical quantification for each endocrine cell type. Data is represented as mean + standard error of the mean, where *** P <0.01. Five hundred cells were quantified from 5 different random field views for each replicate. (C) Reprogrammed cells do not co-express exocrine or mesenchymal markers. Immunocytochemistry in REP+siARX cells for insulin or C-peptide and the exocrine markers CK19 and amylase, and the mesenchymal marker vimentin. Nuclei were counterstained with 4',6-Diamidino-2-Phenylindole, Dihydrochloride (DAPI). Scale bar = 20 μm. (D) Non-reprogrammed cells dedifferentiate towards mesenchymal stromal cells and proliferate in culture. Immunocytochemistry for vimentin, C-peptide and ki67 of untreated exocrine cultures. Nuclei were counterstained with 4',6-Diamidino-2-Phenylindole, Dihydrochloride (DAPI). Scale bar = 20 μm. (TIF) [file pone.0156204.s003.tif]
