# Supplementary material for: Generation of Functional Beta-Like Cells from Human Exocrine Pancreas
Source: PLoS One. 2016 May 31;11(5):e0156204. doi: 10.1371/journal.pone.0156204 (PMC4887015; doi:10.1371/journal.pone.0156204)
Supplement: S1 Table — (DOCX) [file pone.0156204.s004.docx]

S1 Table – List of TaqMan probes used for RT-qPCR analysis.

| **Gene** | **Assay Number** |
| --- | --- |
| PDX1 | Hs00236830_m1 |
| MAFA | Hs01651425_s1 |
| PAX4 | Hs00173014_m1 |
| NGN3 | Hs01875204_s1 |
| NKX6.1 | Hs00232355_m1 |
| NEUROD | Hs00159598_m1 |
| IAPP | Hs00169095_m1 |
| INSULIN | Hs00355773_m1 |
| GLUCAGON | Hs00174967_m1 |
| SOMATOSTATIN | Hs00174949_m1 |
| ARX | Hs00292465_m1 |
| GAPDH | Hs99999905_m1 |
| CHGA | Hs00900375_m1 |
| ABCC8 | Hs01093761_m1 |
| GCK | Hs01564555_m1 |
| PCSK1 | Hs01026107_m1 |
| PCSK2 | Hs00159922_m1 |
| SLC30A8 | Hs00545183_m1 |
