# Supplementary material for: Generation of Functional Beta-Like Cells from Human Exocrine Pancreas
Source: PLoS One. 2016 May 31;11(5):e0156204. doi: 10.1371/journal.pone.0156204 (PMC4887015; doi:10.1371/journal.pone.0156204)
Supplement: S2 Table — (DOCX) [file pone.0156204.s005.docx]

**S2 Table** – List of primary antibodies used for immunocytochemistry.

| **Antigen** | **Host Species** | **Dilution** | **Supplier** |
| --- | --- | --- | --- |
| Pdx1 | Rabbit | 1/800 | Abcam, Cambridge, UK |
| MafA | Rabbit | 1/200 | Santa Cruz Biotechnology, Heidelberg, Germany |
| Arx | Rabbit | 1/200 | Abcam |
| Insulin | Mouse | 1/1000 | Sigma Aldrich |
| C-Peptide | Rabbit | 1/200 | Cell Signalling, Hitchin, UK |
| Somatostatin | Rabbit | 1/50 | Life Technologies |
| Glucagon | Mouse | 1/1000 | Sigma Aldrich |
| Ki67 | Rabbit | 1/200 | Cell Signalling |
| Amylase | Rabbit | 1/500 | Sigma Aldrich |
| CK19 | Mouse | 1/200 | Dako, Glostrup, Denmark |
| Vimentin | Mouse | 1/200 | Dako |
| Nkx6.1 | Rabbit | 1/100 | Novus Biologicals |
